# TLR Antagonism by Sparstolonin B Alters Microbial Signature and Modulates Gastrointestinal and Neuronal Inflammation in Gulf War Illness Preclinical Model

**DOI:** 10.3390/brainsci10080532

**Published:** 2020-08-08

**Authors:** Dipro Bose, Ayan Mondal, Punnag Saha, Diana Kimono, Sutapa Sarkar, Ratanesh K. Seth, Patricia Janulewicz, Kimberly Sullivan, Ronnie Horner, Nancy Klimas, Mitzi Nagarkatti, Prakash Nagarkatti, Saurabh Chatterjee

**Affiliations:** 1 Environmental Health and Disease Laboratory, Department of Environmental Health Sciences, University of South Carolina, Columbia, SC 29208, USA; bosed@email.sc.edu (D.B.); mondala@mailbox.sc.edu (A.M.); psaha@email.sc.edu (P.S.); dkimono@email.sc.edu (D.K.); sutapa@email.sc.edu (S.S.); sethr@mailbox.sc.edu (R.K.S.); 2 Department of Environmental Health, Boston University School of Public Health, Boston, MA 02118, USA; paj@bu.edu (P.J.); tty@bu.edu (K.S.); 3 Department of Health Services Policy and Management, University of South Carolina, Columbia, SC 29208, USA; hornerrd@mailbox.sc.edu; 4 Department of Clinical Immunology, Nova Southeastern University, Fort Lauderdale, FL 33314, USA; nklimas@nova.edu; 5 Miami VA Medical Center, Miami, FL 33125, USA; 6 Department of Pathology Microbiology and Immunology, USC School of Medicine, Columbia, SC 29209, USA; mitzi.nagarkatti@uscmed.sc.edu (M.N.); prakash@mailbox.sc.edu (P.N.); 7 Columbia VA Medical Center, Columbia, SC 29209, USA

**Keywords:** dysbiosis, butyrogenic bacteria, neuroinflammation, nutraceutical, HMGB1, BDNF, astrocytes

## Abstract

The 1991 Persian Gulf War veterans presented a myriad of symptoms that ranged from chronic pain, fatigue, gastrointestinal disturbances, and cognitive deficits. Currently, no therapeutic regimen exists to treat the plethora of chronic symptoms though newer pharmacological targets such as microbiome have been identified recently. Toll-like receptor 4 (TLR4) antagonism in systemic inflammatory diseases have been tried before with limited success, but strategies with broad-spectrum TLR4 antagonists and their ability to modulate the host-microbiome have been elusive. Using a mouse model of Gulf War Illness, we show that a nutraceutical, derived from a Chinese herb Sparstolonin B (SsnB) presented a unique microbiome signature with an increased abundance of butyrogenic bacteria. SsnB administration restored a normal tight junction protein profile with an increase in Occludin and a parallel decrease in Claudin 2 and inflammatory mediators high mobility group box 1 (HMGB1), interleukin-1β (IL-1β), and interleukin-6 (IL-6) in the distal intestine. SsnB also decreased neuronal inflammation by decreasing IL-1β and HMGB1, while increasing brain-derived neurotrophic factor (BDNF), with a parallel decrease in astrocyte activation in vitro. Mechanistically, SsnB inhibited the binding of HMGB1 and myeloid differentiation primary response protein (MyD88) to TLR4 in the intestine, thus attenuating TLR4 downstream signaling. Studies also showed that SsnB was effective in suppressing TLR4-induced nod-like receptor protein 3 (NLRP3) inflammasome activation, a prominent inflammatory disease pathway. SsnB significantly decreased astrocyte activation by decreasing colocalization of glial fibrillary acid protein (GFAP) and S100 calcium-binding protein B (S100B), a crucial event in neuronal inflammation. Inactivation of SsnB by treating the parent molecule by acetate reversed the deactivation of NLRP3 inflammasome and astrocytes in vitro, suggesting that SsnB molecular motifs may be responsible for its anti-inflammatory activity.

## 1. Introduction

Gulf War Illness (GWI) is a chronic, multi-symptomatic disease condition that is known to affect nearly one-third of the veterans who were deployed in the Persian Gulf War of 1990–1991 [1,2]. The symptoms include chronic fatigue, musculoskeletal pain, cognitive deficiencies, respiratory and gastrointestinal disturbances [3]. Exposure to a wide array of toxic chemicals individually or in combination, in the war theatre, was attributed to being the main reason for GWI [3]

Studies show that administration of pyridostigmine bromide (PB) and permethrin (Per) led to gastrointestinal and neurological disturbances in GWI mouse models [4,5]. Our group was the first to report that the GWI mouse model exhibited significant alteration of the gut microbiome, leading to gut leaching, increased endotoxemia, and Toll-like receptor 4 (TLR4) activation which established a mechanistic connection between gastrointestinal and neuroinflammation [6]. We have further confirmed that the administration of the Gulf War (GW) chemicals in mice led to the activation of enteric glial cells through the Toll-like receptor (TLR) pathway [7]. Though there has been extensive research on the underlying pathobiology of GWI over the past decade, yet there are few reported therapeutic approaches specific for GWI that target the Toll-like receptors [8,9,10,11]. 

TLRs play an important role in innate and adaptive immunity [11]. They are expressed in the intestinal epithelial cells (IEC) and detect pathogen-associated molecular patterns (PAMPs) similar to bacterial lipopolysaccharides (LPS) from invading pathogens. TLRs also recognize endogenous signals such as damage-associated molecular patterns (DAMPs), e.g., HMGB1, and recruit adaptor molecules such as myeloid differentiation primary response protein (MyD88) molecules to trigger immunological responses. Activation of TLR4 leads to pro-inflammatory responses causing neuronal injury and secretion of neurotoxic compounds in Alzheimer’s disease (AD) and Parkinson’s disease (PD) [12,13]. We have already established that an increased TLR4 activation is a major cause of the gastrointestinal and neuro-inflammation in GWI [6,12]. Therefore, it led us to investigate the therapeutic effects of TLR2-4 antagonists in our current study [6].

Due to the absence of clinically approved TLR antagonists, we used a potentially established TLR4 antagonist in preclinical studies, Sparstolonin B (SsnB), a natural compound that was isolated from the Chinese herb *Sparganium stoloniferum* [13]. The structural analysis identified SsnB to be a polyphenol having similarities with isocoumarin and xanthone, which attributed to its anti-inflammatory effects. It is reported that SsnB inhibits the TLR4 activation by blocking the binding of TLR4 to MyD88, thereby suppressing/decreasing the nuclear factor kappa-light-chain-enhancer of activated B cells (NF-κB) activation [14,15,16]. SsnB has also been reported to improve outcomes in intracerebral hemorrhage and neuropathic pain in a mice model through a TLR4 dependent manner [17,18]. 

With the above evidence, we hypothesized that the administration of SsnB in the GWI mouse model using PB and Per would significantly modulate gut microbiome, aid in the recovery of GW chemical-induced gut dysbiosis, attenuate gut leaching and expression of DAMPS by antagonizing TLR4 induced inflammation thus improving gut and brain health. In this study, we used an in vivo and in vitro approach to demonstrate the role of SsnB in downregulating TLR4 pathway mediated inflammation in a mouse model of GWI.

## 2. Material and Methods

Per, PB, and Sparstolonin B (SsnB) were purchased from Sigma-Aldrich (St. Louis, MO, USA). Anti-Claudin 2, anti-Occludin, anti-interleukin-6 (IL-6), anti-nod-like receptor protein 3 (NLRP3), anti-Caspase 1, and anti-β-actin were purchased from Abcam (Cambridge, MA USA). Anti-Toll-like receptor 4 (TLR4), anti-S100 calcium binding protein B (S100B), anti-brain derived neurotrophic factor (BDNF), and anti-interleukin-1β (IL-1β) antibodies were purchased from Santacruz Biotechnology (Dallas, TX, USA). Anti-myeloid differentiation primary response protein (MyD88), anti-high mobility group box 1 (HMGB1), anti-apoptosis associated speck-like protein containing a caspase recruitment domain (ASC2), and anti-glial fibrillary acid protein (GFAP) antibodies were purchased from Abclonal Technology (Woburn, MA, USA). Anti-nuclear factor kappa-light-chain-enhancer of activated B cells (NF-κB p65) and anti-phospho NF-κB p65 were purchased from Cell Signaling Technology (Danvers, MA, USA). Species-specific biotinylated conjugated secondary antibody and Streptavidin-horse radish peroxidase (HRP) (Vectastain Elite ABC kit) were purchased from Vector laboratories (Burlingame, CA, USA). Fluorescence conjugated Alexa Fluor secondary antibodies and 4′,6-diamidino-2-phenylindole (DAPI) were purchased from Thermo Fisher Scientific (Rockford, IL, USA). Recombinant mouse HMGB1 was purchased from BioLegend (San Diego, CA, USA). All other chemicals used in this study unless specified were purchased from Sigma. Paraffin embedding of tissues and sectioning on slides were done by AML Laboratories (Jacksonville, FL, USA).

### 2.1. Animals

Adult wild-type male (C57BL/6J) mice of age ten weeks were purchased from Jackson Laboratories (Bar Harbour, ME). The implementation of mice was done in accordance with National Institutes of Health (NIH) guidelines for human care and use of laboratory animals and local IACUC standards. All procedures were approved by the University of South Carolina at Columbia, SC. In accordance with current regulations and guidelines, the Institutional Animal Care and Use Committee (IACUC) at the University of South Carolina has reviewed and approved an action on Animal Use Proposal reference number 101345 (protocol number: 2419-101345-072318) with approval date 11/8/2019. The mouse was housed individually and fed with a chow diet at 22–24 °C with 12 h light/12 h dark cycle. The mice were sacrificed after animal experiments had been completed in seven days. The serum was prepared from freshly obtained blood from mice by cardiac puncture immediately after anesthesia. The serum was preserved at −80 °C until further analysis was performed. The distal part of small intestine and frontal cortex was collected from dissected mice and fixed in 10% neutral buffered formaldehyde or Bouin’s solution (Sigma-Aldrich, St. Louis, MO, USA), respectively and further processed for immunostaining. Fecal pellets were collected from the colon and stored in −80 °C for microbiome analysis.

### 2.2. Rodent Model of Gulf War Illness

Mice were exposed to Gulf War (GW) chemicals pyridostigmine bromide (PB) and permethrin (Per) following established rodent models of Gulf War Illness (GWI) with modifications (7). The acclimatized mice were randomly divided into four groups. The first group was a control (CONT) (*n* = 3) and dosed with vehicle (0.6% dimethyl sulphoxide (DMSO) in a phosphate buffered saline (PBS)). The second group (GW) (*n* = 3) received GW chemicals permethrin (200 mg/kg diluted in DMSO) and pyridostigmine bromide (2 mg/kg diluted in PBS) by oral gavage. The third group (GW+SsnB) (*n* = 3) were treated with PB and Per along with SsnB (3 mg/kg diluted in DMSO) administered intraperitoneally. The fourth group of mice (SsnB) received SsnB through an intraperitoneal route, which served as a control for the GW + SsnB group. GW chemicals and SsnB were dosed every alternate day for a week receiving a total of four doses. 

### 2.3. Cell Culture

#### 2.3.1. Mouse Primary Intestinal Epithelial Cell Culture

Mouse primary intestinal epithelial cells (C576051) were purchased from Cell biologics (Chicago, IL, USA). The cells were maintained according to guidelines provided by Cell biologics. Cells were plated in 12- and 6-well tissue culture plates and allowed to reach 70% confluency in growth. Serum starvation was done using a 1% fetal bovine serum (FBS) in Dulbecco’s modified Eagle’s medium (DMEM) for 18 h and then treated with chemicals. The cells were treated with a vehicle control (0.05% DMSO), mouse recombinant HMGB1 (50 ng/mL), SsnB (10 and 100 μg/mL), and inactivated SsnB (SsnBi) (10 and 100 μg/mL). Inactivation was done by adding an equal concentration of acetate with SsnB [19]. All treatments were for 24 h. After 24 h, the cells were harvested for immunofluorescence staining and protein extraction for Western blot.

#### 2.3.2. Mouse Primary Astrocyte Cell Culture

Primary mouse astrocytes C8-D1A [Astrocyte type I clone] (ATCC CRL-2541) were obtained from ATCC (Manassas, VA, USA). The cells were maintained in DMEM supplemented with 10% FBS. The cells were plated in 12- and 6-well tissue culture plates and growth were allowed until 70% confluency. The cells were sera starved using a 1% fetal bovine serum (FBS) in Dulbecco’s modified Eagle’s medium (DMEM) for 18 h and then treated with chemicals. The cells were treated with a vehicle control (0.05% DMSO), mouse recombinant HMGB1 (50 ng/mL), SsnB (10 and 100 μg/mL), and inactivated SsnB (SsnBi) (10 and 100 μg/mL). All treatments were for 24 h. After 24 h the cells were harvested for immunofluorescence staining. 

### 2.4. Microbiome Analysis

Microbiome analysis was performed using the fecal pellets collected from the mouse after euthanization. Briefly, 100 mg of fecal pellets were used to isolate genomic DNA using the QIamp DNA stool mini kit (Qiagen, Valencia, CA) following the manufacturer’s protocol. Amplification of a 16S rRNA V3-V4 hypervariable region added with an Illumina adapter overhang nucleotide sequence to create a DNA library. The Illumina Miseq platform (San Diego, CA, USA) was used for sequencing. The reads from sequencing were further analyzed by Nephele, an analysis tool by the National Institute of Allergy and Infectious Diseases (NIAID) Office of Cyber Infrastructure and Computational Biology (OCICB) in Bethesda, MD, USA. QIIME FASTQ paired-end with chimera removal, open reference, and the SILVA rRNA database project was used for microbial profiling.

### 2.5. Laboratory Methods

#### 2.5.1. Immunohistochemistry

The distal part of a mouse small intestine and frontal cortex tissues was paraffin embedded and prepared according to the standard protocols and 5 μm thick sections were made. Deparaffinization of the sections were done using the standard protocol. Epitope retrieval of the tissue sections was performed using an epitope retrieval solution and steamer (IHC World, Woodstock, MD, USA). Peroxidase blocking was done using 3% hydrogen peroxide (H_2_O_2_). Blocking was done with 10% serum followed by overnight incubation at 4 °C with primary antibodies against HMGB1, IL-6, IL-1β, and BDNF at 1:200 dilutions. Species specific biotin conjugated secondary antibodies and streptavidin conjugated horseradish peroxidase (HRP) at 1:500 dilutions were used for immunohistochemistry. 3,3’-diaminobenzidine (DAB) (Sigma-Aldrich, St Louis, USA) was used as a chromogenic substrate. Counterstaining was done using Mayer’s hematoxylin solution (Sigma-Aldrich). The stained sections were mounted with Aqua Mount (Lerner Laboratories, Kalamazoo, MI, USA). Sections were observed using the Olympus BX43 and BX63 microscope (Olympus, USA). Morphometric analysis of the images was done using the Cellsens software from Olympus (Center Valley, PA, USA).

#### 2.5.2. Immunofluorescence Staining

Paraffin embedded distal part of mouse small intestine and frontal cortex tissues were deparaffinized using a standard protocol. Epitope retrieval of the tissue sections was performed using an epitope retrieval solution and steamer (IHC World, Woodstock, MD, USA). 10% serum was used for blocking followed by overnight incubation at 4 °C with primary antibodies against Occludin, Claudin 2, TLR4, HMGB1, MyD88, NLRP3, ASC2, Caspase 1, GFAP, and S100B were used at 1:200 dilutions. Species-specific secondary antibodies conjugated with Alexa Fluor (633 red and 488 green) were used at 1:250 dilutions. Mounting of the stained sections was done using a Prolong Diamond antifade reagent with DAPI. Tissue sections were observed under the Olympus BX43 and BX63 fluorescence microscope (Olympus, USA). Morphometric analysis of the images was done using the Cellsens software from Olympus (Center Valley, PA, USA). 

### 2.6. Western Blot

Protein samples were prepared from tissues (distal part of the small intestine and frontal cortex) and cells using a RIPA lysis buffer and quantification were done by the bicinchoninic acid (BCA) assay kit (Thermo Fisher Scientific, IL, USA). Thirty μg of protein from tissue and cell lysates were resolved on Novex 4–12% bis-tris gradient gel and subjected to standard sodium dodecyl sulfate-polyacrylamide gel electrophoresis (SDS-PAGE). The separated proteins were then transferred to the nitrocellulose membrane using the Trans-Blot Turbo transfer system (Bio-Rad, CA, USA). The membrane was stained with Ponceau S followed by blocking in 5% bovine serum albumin (BSA). Incubation with primary antibodies (1:1000 dilution) was done overnight at 4 °C. Species specific anti-IgG secondary antibody conjugated with HRP (1:5000 dilution) was used to tag primary antibody. Blots were developed using ECL Western blotting substrate. The blots were imaged using G: Box Chemi XX6 and densitometric analysis was performed using ImageJ software.

### 2.7. Serum Enzyme linked Immunosorbent Assay (ELISA)

Serum HMGB1 level was estimated with sera from CONT, GW, GW + SsnB, and SsnB mice groups using the ELISA kit from Abclonal Technology (Woburn, MA, USA) following the manufacturer’s protocol.

### 2.8. Statistical Analysis

The in vivo and in vitro experiments were repeated three times and the data were pooled together. We used unpaired t-test and analysis of variance (ANOVA) for statistical analysis followed by Bonferroni post-hoc corrections for comparing between the groups. *p*< 0.05 was considered to be statistically significant.

## 3. Results

### 3.1. SsnB Administration in the GWI Mouse Model Presented a Unique Microbiome Pattern of Higher Abundance of Butyrogenic Bacteria and Prevented Alteration of Tight Junction Protein Levels Claudin 2 and Occludin

**Figure 1:**
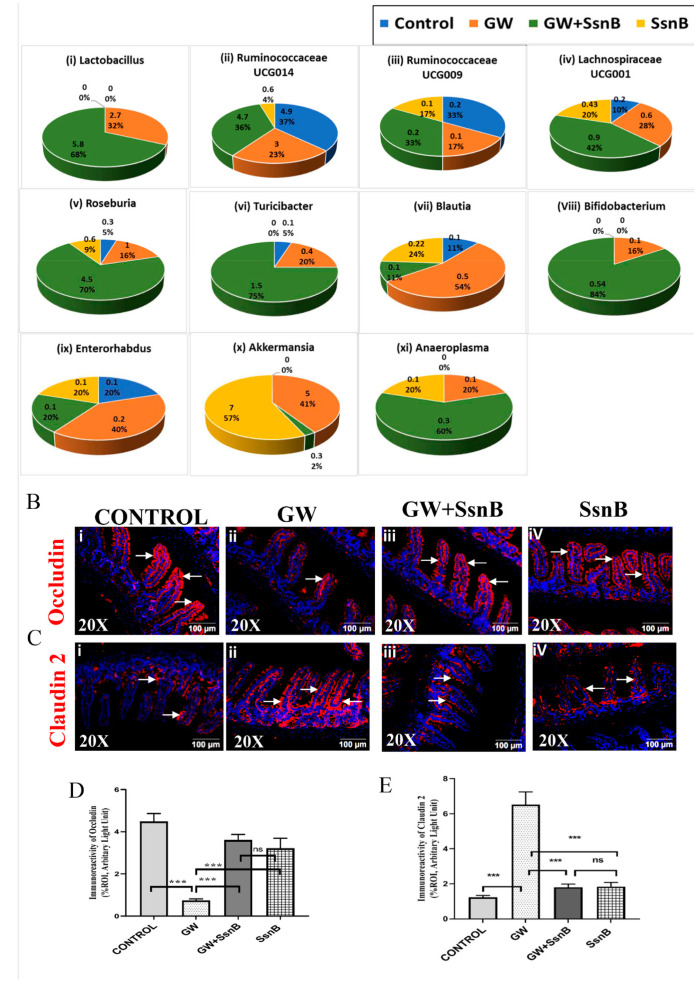
Sparstolonin B (SsnB) restores Gulf War (GW) chemical-induced gut microbial dysbiosis and restores gut leaching. Graphical representation of relative abundance of the microbiome at the genus level that was significantly altered (Figure 1A). The groups compared are CONT (*n* = 3) (wild-type mice which were treated with vehicle), GW (*n* = 3) (GW chemical exposed group), GW + SsnB (*n* = 3) (group exposed with GW chemicals and SsnB), and SsnB (*n* = 3) (group treated with SsnB only). Representative images of the expression of tight junction proteins Occludin (Figure 1B) and Claudin 2 (Figure 1C) were studied by immunofluorescence microscopy. All small intestine sections were stained with a red fluorescent secondary antibody for tight junction protein and were counterstained with DAPI (blue). Immunoreactivity was observed around the epithelial cells of the villi marked by white arrows. Images were taken in 20× magnification. Bar graphs depicting morphometric analysis of Occludin and Claudin 2 immunoreactivity are represented as mean ± standard deviation (SD) of the %ROI (mean value calculated from three separate fields of a section of small intestine) (Figure 1D,E). Significance was analyzed by unpaired T-test where ** *p* < 0.01, *** *p* < 0.001.

To study whether administration of SsnB in the GW mouse model could restore GW chemical (PB and Per) induced microbial dysbiosis, we analyzed mouse fecal pellets using 16S V3-V4 sequencing to study the relative abundance of the microbial load (Figure 1A). The relative abundance of each genus between the four mouse groups are depicted as a percentage, and the overall relative abundance across all the genus identified in microbial analysis is depicted as numerical values. Interestingly, we found that the Firmicutes-Bacteroidetes ratio was increased in GW + SsnB (72%:23%) group compared to the GW chemical exposed group (60%:40%) (Figure S1). At the genus level, we found that the abundance of beneficial microbes belonging to the Firmicutes phyla were increased in the GWI + SsnB group as compared to the GW group. Relative abundance calculated from individual genus OTU percentages of butyrogenic bacteria such as *Lactobacillus, Ruminococcaceae UCG014, Ruminococcaceae UCG009*, *Lachnospiraceae UCG001*, and *Roseburia* which belong to phyla Firmicutes were increased in the GW + SsnB group compared to the GW group. The relative abundance of *Blautia,* however, which also belongs to the butyrogenic bacteria group, was decreased in GW + SsnB compared to the GW group. SsnB administration also increased the relative abundance of genus *Turicibacter* of phylum Firmicutes. The relative abundance of *Enterorhabdus,* a group of bacteria associated with gastrointestinal inflammation and colitis, was found to decrease in the GW + SsnB group compared to GW group of mice [20]. Notably, SsnB administration showed a 16-fold decrease in the relative abundance of *Akkermansia* of phylum Verucomicrobia in GW + SsnB compared to GW group. *Akkermansia* is an inherent gut microbe responsible for maintaining gut barrier function [21]. 

Tight junction proteins are transmembrane proteins that maintain the integrity of the gut barrier against foreign particles and invading pathogens [22]. In the present study, we wanted to examine whether administration of SsnB restored the altered tight junction protein expression due to GW chemical exposure by immunostaining for Claudin 2 and Occludin followed by immunofluorescence microscopy. Results showed a nearly significant increased expression of Claudin 2 and decreased expression of Occludin in the GW group compared to control (Figure 1Ci,ii,Bi,ii,D,E). SsnB administration to GW chemical exposed mice group showed significantly increased expression of Occludin (*p* < 0.01) and significant decreased expression of Claudin 2 (*p* < 0.01) compared to the GW mouse group (Figure 1Biii,iv,Ciii,iv,D,E). 

### 3.2. SsnB Attenuates GW Chemical-Induced Expression of Pro-Inflammatory Cytokines and Damage-Associated Molecular Pattern HMGB1

**Figure 2:**
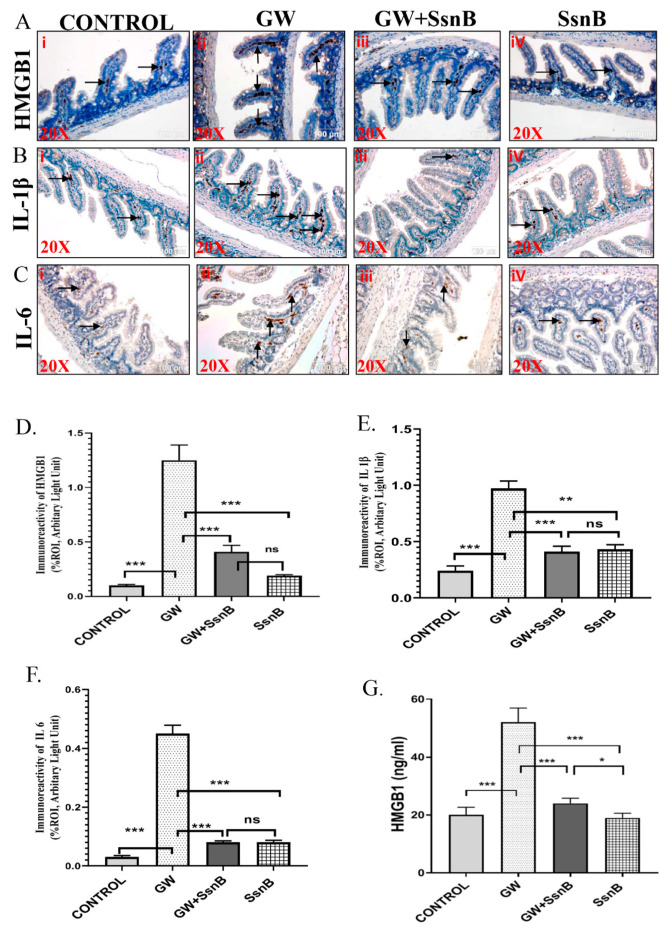
SsnB administration attenuates GW chemical-induced expression of pro-inflammatory cytokines and damage-associated molecular pattern high mobility group box 1 (HMGB1). Representative immunohistochemistry images of small intestine showing immunoreactivity (reactivity was majorly observed in the villi marked with black arrows) for HMGB1 (Figure 2A), IL-1β (Figure 2B), and IL-6 (Figure 2C) in CONT (*n* = 3) (wild-type mice which were treated with vehicle), GW (*n* = 3) (GW chemical exposed group), GW + SsnB (*n* = 3) (group exposed with GW chemicals and SsnB), and SsnB (*n* = 3) (group treated with SsnB only). Images were taken in 20× magnification. Bar graphs depicting morphometric analysis of HMGB1 (Figure 2D), IL-1β (Figure 2E), and IL-6 (Figure 2F) immunoreactivity are represented as mean ± SD of the %ROI (mean value calculated from three separate fields of a section of the small intestine). Significance was analyzed by unpaired T-test where * *p* < 0.05, ** *p* < 0.01, *** *p* < 0.001. Bar graph depicting the serum HMGB1 level at ng/mL (Figure 2G). Significance was analyzed by unpaired T-test where * *p* < 0.05, ** *p* < 0.01, *** *p* < 0.001.

In the present study, we wanted to study if the administration of SsnB could decrease the expression of the pro-inflammatory markers such as IL-1β, IL-6, and DAMPs like HMGB1 that has been shown by us and others as markers for GI inflammation. We probed sections of small intestine with antibodies for HMGB1, IL-1β, and IL-6 by immunohistochemistry to study the immunoreactivity. The expression of HMGB1, IL-1β, and IL-6 was found to be significantly increased in the GW group compared to the control (*p* < 0.01) as observed by the immunoreactivity in the villi of the small intestine (Figure 2Ai,ii,Bi,ii,Ci,ii,D,E,F). However, in the GW + SsnB group, we found that the expression of HMGB1, IL-1β, and IL-6 was significantly decreased (*p* < 0.01) as compared to the GW group (Figure 2Aiii,Biii,Ciii,D,E,F). In a group treated with SsnB only, a similar decrease in expression of pro-inflammatory cytokines was observed (*p* < 0.01) (Figure 2Aiv,Biv,Civ,D,E,F). These results strongly suggest the anti-inflammatory role of SsnB. Results showed a significant increased level of serum HMGB1 in GW chemical exposed mice group (*p* < 0.01) compared to the control (Figure 2G). However, coexposure of SsnB and GW chemicals in mice significantly decreased the level of serum HMGB1 as compared to the GW group (*p* < 0.01) (Figure 2G). 

### 3.3. SsnB Attenuates Gulf War Chemical-Induced TLR4 Activation in the Small Intestine of the GWI Mouse Model

**Figure 3:**
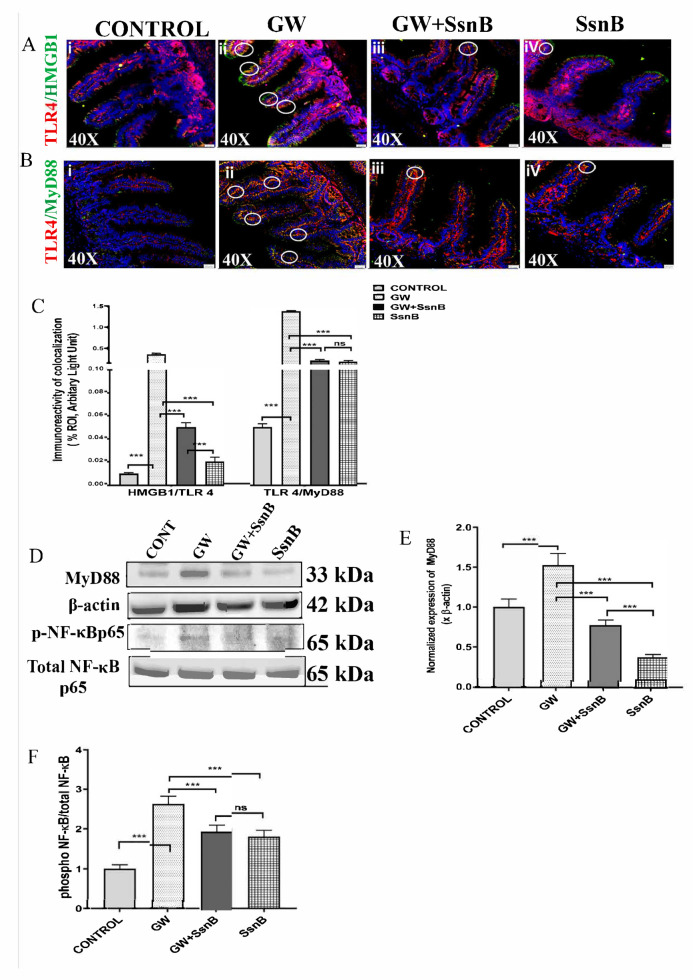
SsnB attenuates Gulf War chemical-induced HMGB1 mediated Toll-like receptor 4 (TLR4) activation in the small intestine of the GWI mouse model. Damage-associated molecular patterns (DAMPs) released due to gut dysbiosis further activates inflammatory signaling pathways through TLR4 receptors. Colocalization study of TLR4 (red)/HMGB1 (green) (Figure 3A) and TLR4 (red)/MyD88 (green) (Figure 3B) was performed using immunofluorescence microscopy in small intestine sections of CONT (*n* = 3) (wild-type mice which were treated with vehicle), GW (*n* = 3) (GW chemical exposed group), GW + SsnB (*n* =3) (group exposed with GW chemicals and SsnB), and SsnB (*n* = 3) (group treated with SsnB only). Colocalizations were observed in the epithelial cells of the villi as yellow dots and were marked in the representative images by white circles. Images were taken at 40× magnification. Bar graphs depicting morphometric analysis of TLR4 /HMGB1 and TLR4/MyD88 (Figure 3C) colocalizations are represented as mean ± SD of the %ROI (mean value calculated from three separate fields of a section of small intestine). Significance was analyzed by unpaired T-test where * *p* < 0.05, ** *p* < 0.01, *** *p* < 0.001. Immunoblot analysis using small intestine tissue lysates was performed to study the role of SsnB in the expression of TLR4 signaling molecules MyD88 and NF-κB (total p65 and phosphorylated p65) (Figure 3D). Bar graph depicting densitometric analysis of MyD88 normalized with β-actin represented mean ± SD (*n* = 3) (Figure 3E). Bar graph depicting densitometric analysis of phosphorylated p65 was normalized with total p65 (Figure 3F). Significance was analyzed by unpaired T-test where * *p* < 0.05, ** *p* < 0.01, *** *p* < 0.001.

In our previous studies in a GWI mouse model, we have found that gut leaching and increased expression of pro-inflammatory cytokines is mechanistically related to TLR4 activation in the small intestine (6). Here, we found that administration of GW chemicals significantly increased expression of HMGB1. HMGB1 is known to be a potent TLR4 ligand that triggers pro-inflammatory signals [23]. Hence, we used immunofluorescence microscopy to study the colocalization of HMGB1/TLR4 by dual labeling HMGB1 (green) and TLR4 (red). Results showed that colocalization events (yellow color) of HMGB1/TLR4 in the epithelial cells surrounding the villi (marked by white circles) was increased in the GW chemical exposed group (GW) compared to the control (*p* < 0.01) (Figure 3Ai,ii,C). Moreover, the colocalization events were significantly decreased in the GW + SsnB treated group compared to the GW group (*p* < 0.01) (Figure 3Aiii,iv,C). 

It is reported that SsnB attenuates TLR4 activation by attenuating the association of adaptor protein MyD88 with TLR4 and consequently the phosphorylation of NF-κB (13). We performed immunofluorescence microscopy to study the interaction of TLR4 with MyD88 through colocalization studies by dual-labeling mouse small intestine sections with TLR4 (red) and MyD88 (green) antibodies. A significant increase in TLR4/MyD88 colocalization events (yellow) was observed (marked in a white circle) in the GW chemical treated group (GW) compared to the control group (CONT) (*p* < 0.01) (Figure 3Bi,ii,C). The colocalization of TLR4/MyD88 was significantly decreased in GW + SsnB and SsnB only groups (*p* < 0.01) (Figure 3Biii,iv,C). Western blot was performed with small intestine tissue lysates from all the groups for protein expression studies. The Western blot results corroborated with our immunofluorescence results, and it showed a significant increase in the expression of MyD88 in GW groups compared to the control. Notably, the expression of MyD88 was markedly decreased in the GW + SsnB group (*p* < 0.01) (Figure 3D,E). Further, we performed Western blot to study the role of SsnB in decreasing the activation of NF-κB. We found that the expression of phosphorylated NF-κB p65 (on comparing with total NF-κB p65) was significantly increased in the GW group compared to the control. However, NF-κB activation was significantly decreased in the GW + SsnB group (*p* < 0.01) (Figure 3D,F). 

### 3.4. Priming with SsnB Attenuates GW Chemical-Induced NLRP3 Inflammasome Activation in the GWI Mouse Model

**Figure 4:**
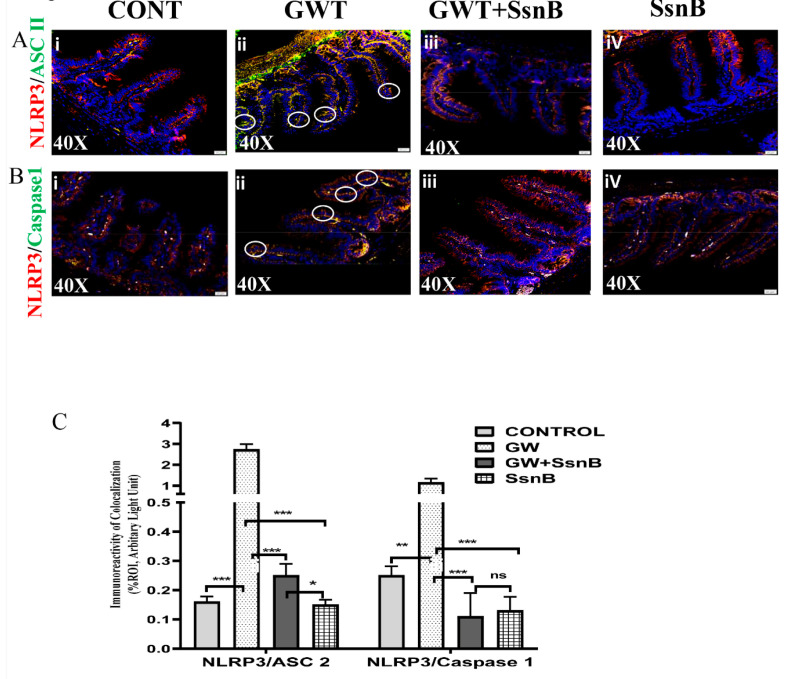
SsnB attenuates GW chemical-induced nod-like receptor protein 3 (NLRP3) inflammasome activation in the GWI mouse model. Activation of TLR4 signaling by HMGB1 may further activate the NLRP3 inflammasome complex, which exacerbates a pro-inflammatory response through secretion of inflammatory cytokines. The colocalization study of NLRP3 (red)/ASC2 (green) (Figure 4A) and NLRP3 (red)/Caspase 1 (green) (Figure 4B) was performed using an immunofluorescence microscopy in small intestine sections of CONT (*n* = 3) (wild-type mice which were treated with vehicle), GW (*n* = 3) (GW chemical exposed group), GW + SsnB (*n* = 3) (group exposed with GW chemicals and SsnB), and SsnB (*n* = 3) (group treated with SsnB only). Colocalizations were observed in the epithelial cells of the villi as yellow dots and are marked in the representative images by white circles. Images were taken at 40× magnification. Bar graphs depicting morphometric analysis of NLRP3/ASC2 and NLRP3/Caspase 1 (Figure 4C) colocalizations are represented as mean ± SD of the %ROI (mean value calculated from three separate fields of a section of the small intestine). Significance was analyzed by unpaired T-test where * *p* < 0.05, ** *p* < 0.01, *** *p* < 0.001.

The secretion of pro-inflammatory cytokines such as IL-1β occurs through the activation of cytosolic pattern recognition receptors (PRRs) called inflammasomes [24]. NLRP3 is one such inflammasome that can be activated by DAMPs like HMGB1 [25]. In order to study NLRP3 activation in the GW chemical exposed group and the potential of SsnB in downregulating its activation, immunofluorescence microscopy was used. Briefly, we dual-labeled mouse small intestine section with NLRP3 (red) and its adaptor protein ASC2 (green) antibodies. Significantly increased colocalization events of NLRP3-ASC2 was observed (observed as yellow dots on the epithelial cells in villi, marked by white circle) in the GW group compared to the control group (*p* < 0.01) (Figure 4Ai,ii,C) confirming NLRP3 inflammasome activation in the intestine of GW mouse groups. Consequently, the colocalization events were considerably decreased in the GW + SsnB and group treated with SsnB only (*p* < 0.01) (Figure 4Aiii,iv,C). To further confirm the decrease of NLRP3 inflammasome activation by SsnB, co-localization events of NLRP3 (red), and Caspase 1 (green) was studied in the intestine sections. Results showed significantly decreased colocalization events of NLRP3/Caspase 1 in the GW + SsnB group and groups treated with SsnB only as compared with the GW group (*p* < 0.01) (Figure 4B,C).

### 3.5. SsnB Attenuates HMGB1 Induced NLRP3 Activation in Primary Mouse Intestinal Epithelial Cells

**Figure 5:**
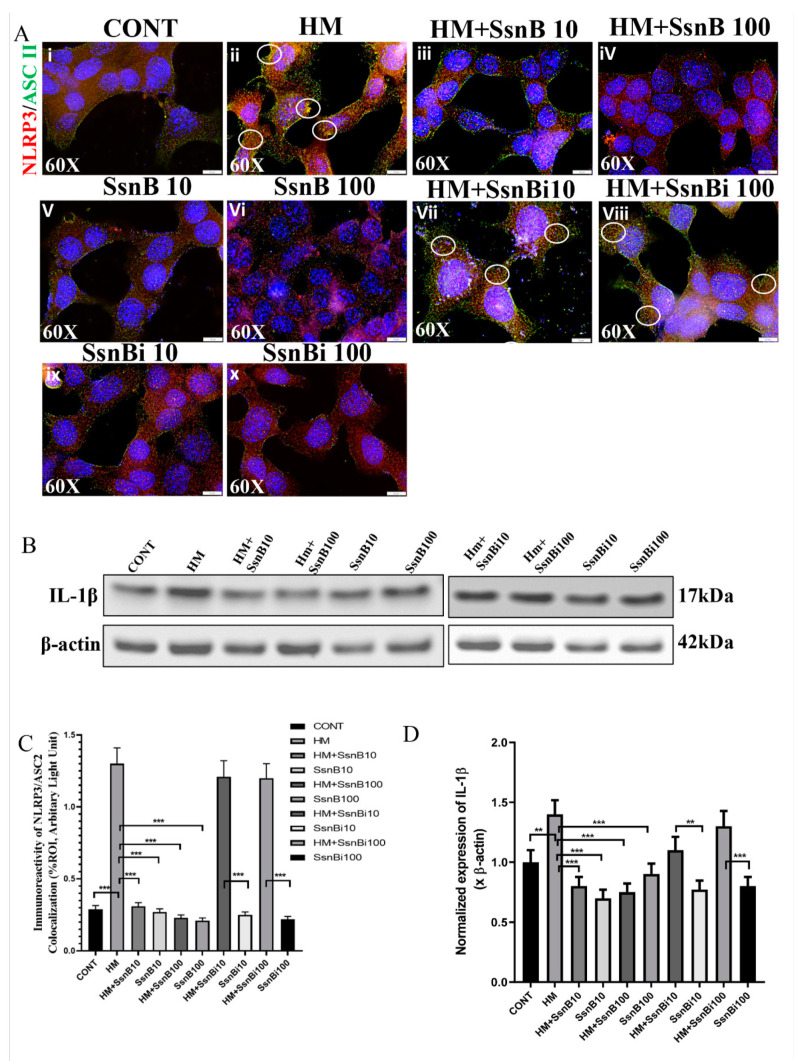
SsnB attenuates HMGB1 induced NLRP3 inflammasome activation in primary mouse intestinal epithelial cells. Mouse primary intestinal epithelial cells were treated with HMGB1 (HM), HMGB1 with SsnB at 10 μg/mL (HM + SsnB10) and 100 μg/mL (HM + SsnB100) concentrations and only SsnB at 10 μg/mL (SsnB10) and 100 μg/mL (SsnB100) concentrations. To confirm the role of SsnB in attenuating HMGB1 induced NLRP3 activation, the cells were further treated with inactivated or pseudo-SsnB along with HMGB1 at 10 μg/mL (HM + SsnBi10) and 100 μg/mL (HM + SsniB100) concentrations. Inactivated SsnB at the two concentrations (SsnBi10 and SsnBi100, respectively) were used as controls to compare with HM + SsnBi10 and HM + SsniB100. The colocalization study of NLRP3 (red)/ASC2 (green) (Figure 5A) was performed using immunofluorescence microscopy. Colocalizations were observed in the cytoplasmic region of the cells as yellow dots and are marked in the representative images by white circles. Images were taken at 60× magnification. Bar graphs depicting morphometric analysis of NLRP3/ASC2 (Figure 5C) colocalizations are represented as mean ± SD of the %ROI (mean value calculated from three separate fields of a sections). Significance was analyzed by unpaired T-test where * *p* < 0.05, ** *p* < 0.01, *** *p* < 0.001. Immunoblot analysis using mouse primary intestinal epithelial cell lysates was performed to further study the expression of IL-1β (Figure 5B). Bar graphs depicting densitometric analysis of immunoblots represented mean ± SD (*n* = 3), which were normalized with β-actin. Significance was analyzed by unpaired T-test where ** *p* < 0.01, *** *p* < 0.001 (Figure 5D).

Previous studies have shown that DAMPs like HMGB1 induced NLRP3 inflammasome activation in the small intestine [26]. To confirm that HMGB1 induced NLRP3 activation, we performed an in vitro study using primary mouse intestinal epithelial cells and performed immunofluorescence microscopy by dual-labeling cells with NLRP3 (red) and ASC2 (green) antibody. Results showed that colocalization events were increased in cells treated with HMGB1 (observed as yellow dots around the nucleus of the cells in the cytoplasm and marked with a white circle) compared to the control (CONT) group (*p* < 0.01) (Figure 5Ai,ii,C). Notably, the events of colocalization were markedly decreased in groups treated separately with two different concentrations of SsnB (10 and 100 μg/mL) and HMGB1 compared to cells treated with HMGB1 alone (*p* < 0.01) (Figure 5Aiii,iv,v,vi,C). We wanted to confirm the role of SsnB in decreasing the NLRP3 expression. We inactivated SsnB by acetylation and treated primary mouse intestinal epithelial cells with HMGB1 and the pseudo form or the inactivated SsnB (at 10 and 100 μg/mL) and dual labeled for NLRP3 (red) and ASC2 (green) antibodies. We found that cells treated with the inactivated SsnB along with HMGB1 had increased colocalization events of NLRP3/ASC2 comparable to the cells treated with HMGB1, only suggesting that SsnB was solely responsible for the inhibition (*p* < 0.01) (Figure 5Avii.viii,ix,x,C). We further confirmed our results by probing for IL-1β with cell lysates treated with HMGB1, HMGB1, and SsnB (at two different concentrations) and HMGB1 with an inactivated SsnB (Figure 5B,D) by Western blot.

### 3.6. SsnB Improves GW Chemical-Induced Neuroinflammation in Mouse GWI Model

**Figure 6:**
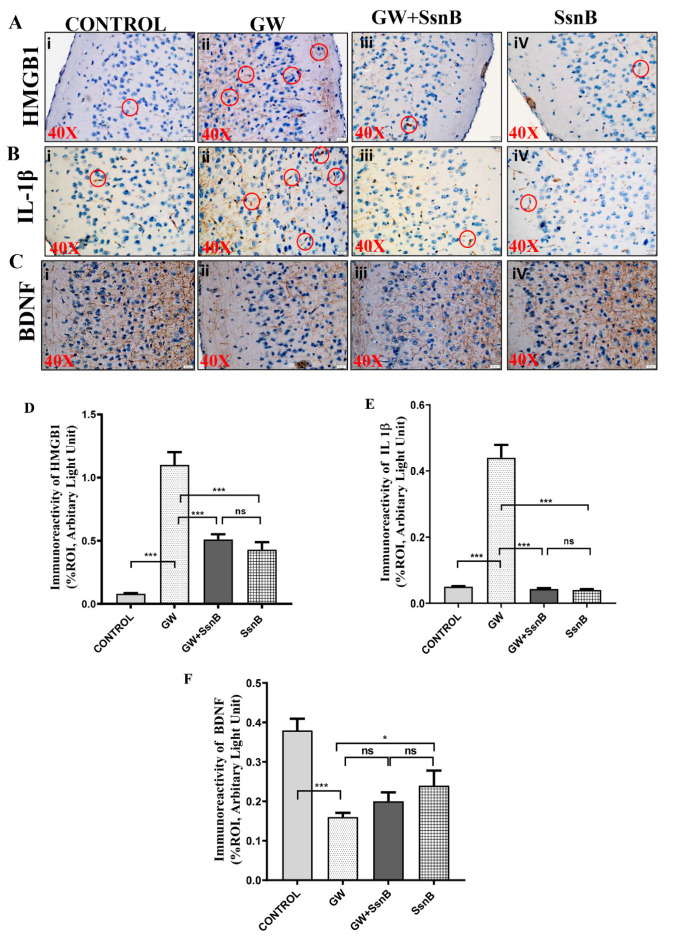
Administration of SsnB improves GW chemical-induced neuroinflammation in the mouse GWI model. Representative immunohistochemistry images of frontal cortex region showing immunoreactivity (marked with red circles) for HMGB1 (Figure 6A), IL-1β (Figure 6B), and neuronal plasticity marker BDNF (Figure 6C) in CONT (*n* = 3) (wild-type mice which were treated with vehicle), GW (*n* = 3) (GW chemical exposed group), GW + SsnB (*n* = 3) (group exposed with GW chemicals and SsnB), and SsnB (*n* = 3) (group treated with SsnB only). Images were taken in 40× magnification. Bar graphs depicting morphometric analysis of HMGB1 (Figure 6D), IL-1β (Figure 6E), and BDNF (Figure 6F) immunoreactivity are represented as mean ± SD of the %ROI (mean value calculated from three separate fields of a section of frontal cortex). Significance was analyzed by unpaired T-test where * *p* < 0.05, ** *p* < 0.01, *** *p* < 0.001.

Studies conducted by us and others have reported that GW chemical exposure led to neuroinflammation with increased expression of pro-inflammatory cytokines in a murine model of GWI [6,10]. We wanted to study whether SsnB induced improvement of gut health could consequently improve GW chemical-induced neuroinflammation. Immunohistochemistry was performed with HMGB1, IL-1β, and BDNF antibodies in mouse brain sections and results showed that expression of HMGB1 and IL-1β were significantly increased in the GW group (as observed by immunoreactivity in the frontal cortex region of the brain) compared to the control group (*p* < 0.01) (Figure 6Ai,ii,Bi,ii,D,E). Moreover, the expression of HMGB1 and IL-1β was significantly decreased in the GW + SsnB group and group treated with SsnB only (*p* < 0.01) (Figure 6Aiii,iv, Biii,iv,D, E). We also found that expression of BDNF, an important marker for neuronal plasticity [27] was markedly decreased in the GW chemical treated group compared to the control group (*p* < 0.01) (Figure 6Ci,ii,F). However, the expression of BDNF was found to be increased in GW + SsnB (not statistically significant) and in the group treated with SsnB only (*p* < 0.05) (Figure 6Ciii,iv,F). 

### 3.7. SsnB attenuates GW Chemical-Induced TLR4 Activation and Inflammation Astrocytes of the GWI Mouse Model.

**Figure 7:**
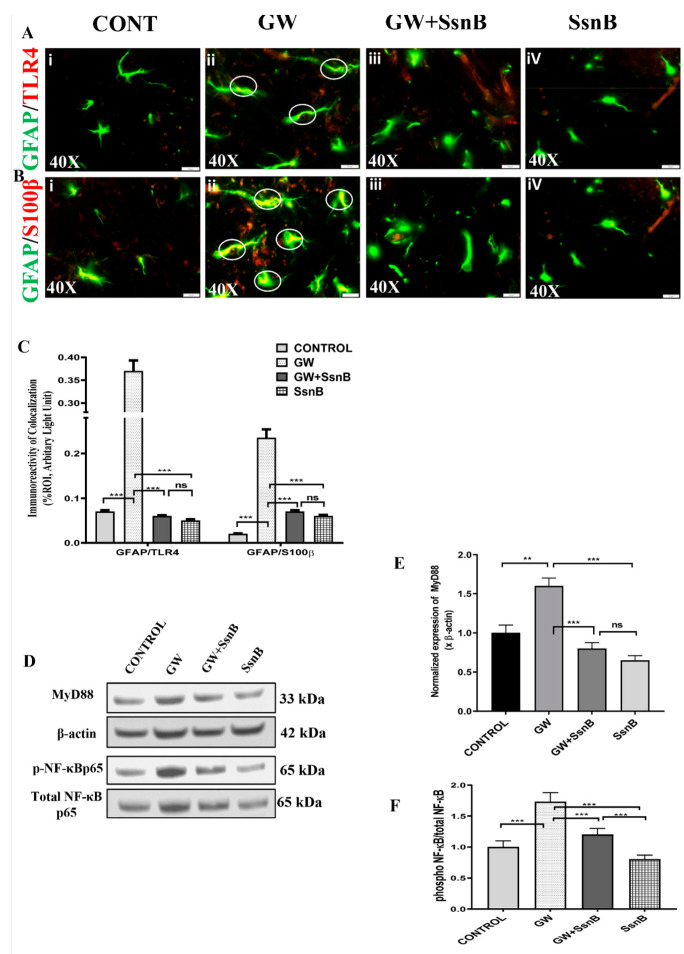
Treatment with SsnB attenuates GW chemical-induced HMGB1 mediated TLR4 activation and inflammation in the hippocampal region in the GWI mouse model. Increased serum expression of HMGB1 in the GW chemical treated mouse group suggests that HMGB1 may cause inflammation in ectopic sites. Colocalization study of TLR4 (red)/GFAP (green) (Figure 7A) and S100B (red)/GFAP (green) (Figure 7B) was performed using immunofluorescence microscopy in the hippocampal region in the brain of CONT (*n* = 3) (wild-type mice which were treated with vehicle), GW (*n* = 3) (GW chemical exposed group), GW + SsnB (*n* = 3) (group exposed with GW chemicals and SsnB), and SsnB (*n* = 3) (group treated with SsnB only). Colocalizations were observed in the astrocytes (labeled with GFAP) as yellow dots and are marked in the representative images by white circles. Images were taken at 40× magnification. Bar graphs depicting morphometric analysis of TLR4/GFAP and S100B/GFAP (Figure 7C) colocalizations are represented as mean ± SD of the %ROI (mean value calculated from three separate fields of a section of the small intestine). Significance was analyzed by unpaired T-test where * *p* < 0.05, ** *p* < 0.01, *** *p* < 0.001. Immunoblot analysis was performed using protein extracted from frontal cortex lysates to further study the role of SsnB in the expression of TLR4 signaling molecules MyD88, NF-κB (total p65 and phosphorylated p65) (Figure 7D). Bar graphs depicting densitometric analysis of MyD88 immunoblot normalized with β-actin represented mean ± SD (*n* = 3) (Figure 7E). Bar graphs depicting densitometric analysis of phosphorylated p65 normalized with total p65 (Figure 7F). Significance was analyzed by unpaired T-test where ** *p* < 0.01, *** *p* < 0.001.

Astrocytes have been reported to play an essential role in the regulation of neuroinflammation in the brain. This occurs through the participation of TLR4 receptors that are shown to have a pivotal role in the activation of astrocytes [28]. In this study, we wanted to examine whether SsnB can downregulate GW chemical-induced expression of TLR4 in astrocytes. On dual-labeling mouse brain sections with GFAP (green) and TLR4 (red) antibodies, we found that TLR4/GFAP colocalization events (observed in the hippocampal region of the brain) were significantly decreased in the GW + SsnB group as compared with the GW group (*p* < 0.01) (Figure 7Aiii,iv,C). Notably, the highest colocalization events were observed in the GW group when compared to the control (*** *p* < 0.001) (Figure 7Ai,ii,C). In order to study astrocyte inflammation, we performed dual-labeling of GFAP (green) with S100B (red) antibodies. Results showed that GFAP/S100B colocalization events were significantly increased in the GW group compared to the control (*** *p* < 0.001) (Figure 7Bi,ii). In the GW + SsnB group, the events of colocalization were significantly decreased compared to the GW group (*** *p* < 0.001) (Figure 7Biii,iv,C). Further, we went on to study the mechanism through which SsnB attenuates inflammation in astrocytes. Western blot analysis for Myd88 and NFKB was performed using mouse brain lysates. Our results showed that the expression of Myd88 was decreased in the GW + SsnB group compared to the GW group, whereas the expression of phosphorylated NF-κB was significantly decreased in the GW + SsnB group as compared to the GW group (*p* < 0.01) (Figure 7D,E,F). 

### 3.8. SsnB Suppresses HMGB1 Induced Expression of GFAP/S100B in Mouse Primary Astrocytes

**Figure 8:**
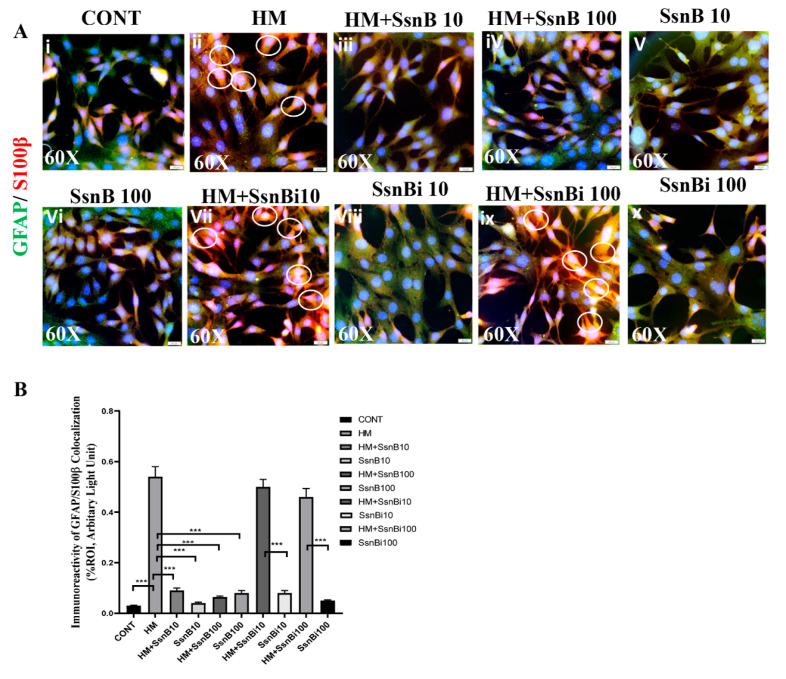
SsnB reduces HMGB1 induced expression of inflammatory markers GFAP/S100B in mouse primary astrocytes. Mouse primary astrocyte cells were treated with HMGB1(HM), HMGB1 with SsnB at 10 μg/mL (HM + SsnB10) and 100 μg/mL (HM + SsnB100) concentrations and only SsnB at 10 μg/mL (SsnB10) and 100 μg/mL (SsnB100) concentrations. The cells were also treated with inactivated or pseudo-SsnB along with HMGB1 at 10 μg/mL (HM + SsnBi10) and 100 μg/mL (HM + SsniB100) concentrations. Inactivated SsnB at the two concentrations (SsnBi10 and SsnBi100, respectively) were used as controls to compare with HM + SsnBi10 and HM + SsniB100. Immunofluorescence microscopy was used to study the inflammation induced by HMGB1 by dual-labeling the cells with S100B (red)/GFAP (green). Colocalizations were observed in the cytoplasmic region of the cells as yellow dots and are marked in the representative images by white circles (Figure 8A). Images were taken at 60× magnification. Bar graphs depicting morphometric analysis ofS100B/GFAP (Figure 8B) colocalizations are represented as mean ± SD of the %ROI (mean value calculated from three separate fields of a section). Significance was analyzed by unpaired T-test where ** *p* < 0.01, *** *p* < 0.001.

In this study, we have found that the concentration of serum HMGB1 was highest in the GW chemical treated group as compared to control and GW + SsnB groups as quantified by ELISA. This led us to hypothesize that the circulatory HMGB1 may cause inflammation in distant sites from the source, i.e., the small intestine. We treated mouse primary astrocytes with HMGB1 and SsnB (at 10 and 100 μg/mL concentration) and performed immunofluorescence microscopy by dual-labeling with GFAP (green) and S100B (red) antibodies. We found that cells treated with HMGB1 and SsnB had marked decreased by in colocalization events with GFAP/S100B as compared to cells treated with HMGB1 only (*p* < 0.01) (Figure 8i-vi,B). Cells treated with HMGB1 and inactivated SsnB (at 10 and 100 μg/mL concentration) gave results similar to cells treated with HMGB1 (*p* < 0.05) (Figure 8vii,viii,ix,x,B). 

## 4. Discussion

Our results showed that SsnB administration, a known TLR4 antagonist in mice that were pre-exposed to GW chemicals, presented a unique microbiome signature when compared to GW chemical-induced alteration of gut microflora. SsnB increased the relative abundance of butyrogenic bacteria known for their role in improving immune and gut health when compared to the GW chemical treated mouse group. The relative abundance of *Anaeroplasma,* a group of commensal bacteria that maintains the mucosal IgA level strengthening the gut barrier, was increased by three folds in the mouse group treated with SsnB and GW chemicals (GW + SsnB) compared to the GW chemical treated mouse group [29]. Dietary supplements that include nutraceuticals embedded in diets or consumed as a supplement have shown significant therapeutic benefits against chronicity of multisymptomatic gastrointestinal disease [30]. We and others have shown that dietary short-chain fatty acids or diet per se play a role in GWI pathogenesis [9,31]. The probiotic and pro-butyrogenic functions of SsnB that was observed in the mouse model was significant and a novel outcome. SsnB was introduced in the mouse model through the intraperitoneal route. Hence, it may be the inherent anti-inflammatory property of SsnB primarily by preventing TLR4 mediated signaling that decreases the GW chemical-induced inflammation in the gut leading to the restoration of gut dysbiosis and not its direct actions on the microflora per se. The increase in butyrogenic bacteria may also be due to a strong anti-inflammatory microenvironment in the gut epithelial cells that may differentially regulate colonization of bacterial species.

Interestingly, we found that the relative abundance of *Akkermansia* was decreased in the mouse group treated with SsnB and GW chemicals (GW + SsnB) compared to the GW chemical exposed group of mice. *Akkermansia* is a part of gut commensal microbiota that also plays an essential role in maintaining the metabolic and immunological functions along with maintaining the level of tight junction proteins in the healthy human gut [32]. It has been established that *Akkermansia* imparts its protective functions through TLR2 and TLR4 activation by its pili-like protein Muc-T and Amuc_1100, thereby secreting anti-inflammatory cytokine interleukin-10 (IL-10) [21]. In our study, blocking TLR4 activation by SsnB might have resulted in the decrease of *Akkermansia* population. However, the mechanism needs to be further investigated, and our study is limited to define the cause of its decrease. Our observation of a decrease in Genus OTU percent of *Akkermansia* is not reflective of depletion of that species, rather it only shows a comparative decrease vis a vis other groups. The decrease in *Akkermansia* species might also be explained by the above rationale of restrictive colonization of this species due to a strong anti-inflammatory effect of SsnB rather than the direct effect of the drug on the growth of the bacteria in the gut lumen though the above argument is speculative at this point. The above observation of a decrease in genus OTU percent for *Akkermansia* perhaps also highlights the importance of intact TLR4 signaling in the gut for launching a robust immune response. A complete blockage of TLR4 signaling in the gut by TLR4 antagonists such as SsnB might not augur well for the overall gut health though the anti-inflammatory effect of SsnB appears to attenuate the inflammation associated with GWI. An altered *Akkermansia* species abundance may subject the GWI-mouse for yet unknown gut-related health risks though its GWI-related inflammation may have been cured.

Results showed that the expression of tight junction proteins that were altered on GW chemical exposure resulting in a possible gut leaching observed in a similar study were restored to control levels following SsnB administration. Apparently, SsnBs role as an antagonist to TLR4 and its effect in decreasing Claudin 2 and increasing Occludin might have resulted from blockage of TLR4 downstream signaling in the epithelial cells of the gut. Claudin 2, a gut barrier protein is known to be upregulated in inflammation and has a pronounced role in causing gut barrier integrity loss [33]. Studies have shown that TLR4 signaling-induced pro-inflammatory cytokines such as tumor necrosis factor-α (TNF-α), IL-6, and IL-17 cause an increase in the levels of Claudin 2 via the phosphoinositide 3-kinase (PI3 Kinase) pathway [34]. A strong anti-inflammatory role of SsnB by blocking TLR4 signaling might have caused the observed decrease in Claudin 2 in our model. However, other studies show that TLR4 is an important molecule in recognizing microbial motifs and may be important for maintaining gut barrier integrity as reviewed elsewhere by other mechanisms not related to the transcription of gut barrier proteins [35]. Our observed effects of SsnB may be primarily through TLR4 antagonism, but other cellular functions of this molecule cannot be ruled out. 

We further went on to study the TLR4 activation pathway that plays a pivotal role in gastrointestinal inflammation in GWI. Studies show that HMGB1 released by the damaged intestinal epithelial cells may act as a ligand for TLR4 thereby activating a signaling cascade that further exacerbates gastrointestinal inflammation in IBD conditions [36,37]. Our results showed that GW chemical treated mice had an increased binding of HMGB1-TLR4, as seen in fluorescent microscopy that was decreased in mice groups that were exposed to GW chemicals and received SsnB. SsnB has already been established as a molecule that downregulates TLR4 activation by reducing the binding of MyD88 and decreasing NF-κB p65 activation [13]. In our results, we found that SsnB decreased TLR4-MyD88 binding and NF-κB p65 activation in mice groups administered with GW chemicals and SsnB compared to the GW chemical exposed mice group. Activation of TLR4 by HMGB1 may further activate cytosolic NLRP3 inflammasomes by recruiting adaptor protein ASC2 and Caspase 1 in intestinal epithelial cells that lay the groundwork for secretions of pro-inflammatory cytokines IL-1β and IL-18 [26]. Our study further reports that SsnB treatment with GW chemicals decreased TLR4 mediated NLRP3 activation through a decrease in NLRP3-ASC2 and NLRP3-Caspase1 complex formation in intestinal epithelial cells. NLRP3-inflammasomes are pivotal in disease-specific inflammatory pathways [38]. We recently reported their role in GWI and inflammation persistence (Kimono D et al., Neurosci Insights-In press). This was confirmed by an in vitro study by priming mouse primary intestinal cells with HMGB1 and treating with two different concentrations of SsnB (10 and 100 μg/mL) where NLRP3 activation was significantly decreased by SsnB. To further confirm the role of SsnB in the mechanistic pathways in vitro, we degraded the functional domain of SsnB by acetylation of the primary moiety and generating an inactivated or pseudo-SsnB. We found that the treatment of cells with inactivated SsnB with HMGB1 produced results similar to HMGB1-only treated cells. The above data confirmed SsnB to be the sole compound responsible for NLRP3 inactivation. However, the present study is limited in its more in-depth mechanistic approach about the structure of SsnB since it does not probe or characterize of the chemical moiety of acetylated SsnB. Notably, gastrointestinal inflammation is also triggered by activation of the enteric glial cells as shown by us previously [7]. Though we did not explore the role of SsnB in modulating the glial cells that are an integral part of the enteric nervous system, it is worth studying the TLR4 antagonism in these pathways [10,36]. 

Gulf War Illness is a neuroimmune disease [37]. Neuroinflammation and cognitive disorders induced likely by GW chemicals are observed to be the most commonly occurring symptoms of veterans with GWI [3,39]. We and others have found that neuroinflammation in GWI mouse models causes increased expression of pro-inflammatory cytokine such as IL-1β, IL-6, TNF-α, and DAMPs like HMGB1 in the brain, damaged blood-brain barrier (BBB) through alteration of tight junction protein, decreased expression of neuronal plasticity markers, and activation of TLR4 in the brain [6,10,40,41]. Results showed that SsnB exposure significantly decreased elevated expression of HMGB1 and IL-1β in GW groups. Expression of BDNF was also found to increase with the SsnB treatment though not significantly. We also found that SsnB reversed the GW chemical-induced inflammatory phenotype of brain astrocytes through a decrease in TLR4 activation and expression of inflammatory molecule S100B. An increased level of circulatory HMGB1 observed in the serum of GW mice may act as one of the mediators crossing the damaged blood-brain barrier and causing ectopic inflammation. Release of exosomes during gut dysbiosis containing HMGB1 may be the possible means for the transfer of the above DAMPs across BBB [42]. However, the present study could not decipher whether TLR4 activation occurrs by circulatory HMGB1 or endogenous HMGB1 secreted by neighboring damaged tissues such as activated microglia The interconnectivity of the astrocytes with other neurons is extensive, impacting significantly the neurovascular units of the brain [43]. It has been reported that the downregulation of NF-κB activation in astrocytes decreases inflammation and aids in recovery from spinal cord injury [43]. The results in vitro of SsnB significantly decreasing HMGB1-TLR4 pathway activation via downregulation of NF-Kb phosphorylation is proof of its role in blocking the TLR4 signaling-induced inflammation. 

The above experiments in deciphering the role of SsnB was carried out in an in vivo model of GWI pathology. The model is an adaptation from various other rodent models of GWI pathogenesis that mainly rely on GW-related chemical exposures and consumption of pharmaceuticals prescribed to the troops at that time. The prescribed drug used in our model is Pyridostigmine Bromide (PB). It is worth noting that not one model is a perfect platform for investigating GWI pathogenesis since exposures during that time period varied widely [5,36,40,41]. The use of an insecticide such as Permethrin is representative of the harmful effects of these classes of compounds [3]. It is important that advancing GWI pathogenesis research and testing preclinical efficacies should include multiple GWI models. Further exposure routes for insecticides such as Permethrin should also be tried intranasally to better model the effects in GWI. 

## 5. Conclusions

The evidences presented in this study both in vivo and in vitro support a strong therapeutic role of SsnB, a relatively non-toxic nutraceutical in GW chemical-induced neuroinflammation and can be rapidly cleared for a clinical trial and approved for treatment in veterans with GWI.

## Supplementary Materials

The following are available online at https://www.mdpi.com/2076-3425/10/8/532/s1. Figure S1: Graphical representation of percentage relative abundance of microbiome at phylum level (bacteroidetes and firmicutes) that were significantly altered. The groups compared are CONT (*n* = 3) (wild-type mice which were treated with vehicle), GW (*n* = 3) (GW chemical exposed group), GW + SsnB (*n* = 3) (group exposed with GW chemicals and SsnB), and SsnB (*n* = 3) (group treated with SsnB only).
