# Supplementary figures and images for: TLR Antagonism by Sparstolonin B Alters Microbial Signature and Modulates Gastrointestinal and Neuronal Inflammation in Gulf War Illness Preclinical Model
Source: Brain Sci. 2020 Aug 8;10(8):532. doi: 10.3390/brainsci10080532 (PMC7463890; doi:10.3390/brainsci10080532)

**Supplementary Fig. 1**

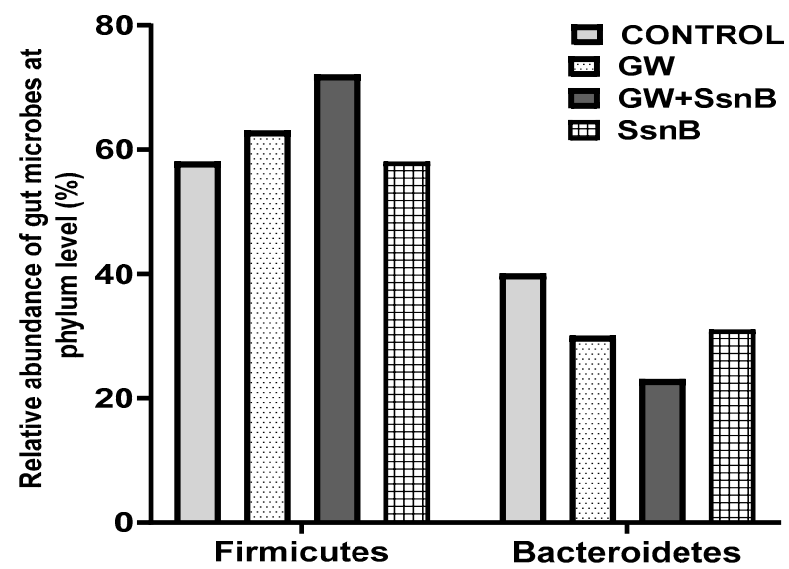

Supplement: Supplementary file 1 [file brainsci-10-00532-s001.pdf]
